# *MET* amplification assessed using optimized FISH reporting criteria predicts early distant metastasis in patients with non-small cell lung cancer

**DOI:** 10.18632/oncotarget.24430

**Published:** 2018-02-07

**Authors:** Lianghua Fang, Hui Chen, Zhenya Tang, Neda Kalhor, Ching-Hua Liu, Hui Yao, Shimin Hu, Pei Lin, Jin Zhao, Raja Luthra, Rajesh R. Singh, Mark J. Routbort, David Hong, L. Jeffrey Medeiros, Xinyan Lu

**Affiliations:** ^1^ Department of Hematopathology, The University of Texas MD Anderson Cancer Center, Houston, TX, USA; ^2^ Department of Pathology, The University of Texas MD Anderson Cancer Center, Houston, TX, USA; ^3^ Department of Bioinformatics and Computational Biology, The University of Texas MD Anderson Cancer Center, Houston, TX, USA; ^4^ Department of Investigational Cancer Therapeutics, The University of Texas MD Anderson Cancer Center, Houston, TX, USA; ^5^ Department of Oncology, Jiangsu Province Hospital of Traditional Chinese Medicine, Nanjing, Jiangsu, China; ^6^ Department of Clinical Laboratory, Hunan Cancer Hospital & The Affiliated Cancer Hospital of Xiangya School of Medicine, Central South University, Changsha, Hunan, China; ^7^ Department of Pathology, Northwestern University Feinberg School of Medicine, Chicago, IL, USA

**Keywords:** MET gene amplification, MET FISH reporting criteria, non-small cell lung cancer, distant metastasis

## Abstract

To investigate the prognostic impact of *MET* copy number (*MET*-CN) in patients with non-small cell lung cancer (NSCLC), we retrospectively reviewed clinical and pathologic data of NSCLC patients whose tumors were assessed for *MET*-CN using fluorescence *in situ* hybridization (FISH). We correlated *MET*-CN status with patient overall survival (OS) and optimized *MET*-FISH reporting criteria. The study group included 384 patients with NSCLC of which 88% were adenocarcinoma and 55.7% of patients had distant metastases. There were 170 patients with stages I-III and 214 patients with stage IV disease. Based on the *MET*-CN and *MET*/CEP7 ratio the patients were classified into 3 categories: *MET*-amplification (*MET*amp): *MET*/CEP7 ≥ 2 or *MET*-CN ≥ 5; *MET*-CN-gain (*MET*cng): *MET*-CN ≥ 4 to < 5; and *MET*-negative (*MET*neg): *MET*-CN < 4. *MET*amp was associated with high fatality (*P*=.036) and stage IV tumors (*P*=.038). In patients with stages I-III NSCLC, patients in the *MET*amp category had the shortest OS (*P*=.015) and more often developed distant metastases within 1 year (*P*=.004). In patients with stage IV tumors, *MET*amp did not further impact the OS. Patients in the *MET*cng category had the longest OS (*P*=.053). Multivariate analysis confirmed *MET*amp to be an independent high-risk factor (HR 3.26; *P*=.026) and predicted earlier progression to distant metastasis (HR 4.86; *P*=.001). In conclusion, we suggest that the *MET*-FISH criteria presented optimizes risk stratification by defining 3 categories of NSCLC patients. *MET*amp is an independent risk factor predicting early distant metastasis and patients with *MET*cng could represent a lower-risk group.

## INTRODUCTION

Chromosomal aneuploidy or somatic copy number alterations (SCNAs) are frequently observed in malignant neoplasms including lung cancers and have been proposed to drive tumorigenesis or treatment resistance [1, 2]. SCNAs such as amplification of the mesenchymal-epithelial transition factor gene (*MET*) which resides on chromosome 7q31, can be detected conveniently by using fluorescence *in situ* hybridization (FISH). *MET* amplification (*MET*amp) has been associated with shorter overall survival (OS) in patients with non-small cell lung cancer (NSCLC) [3–6]. Therefore, *MET* is considered a potentially targetable oncogenic driver [7, 8]. Recent studies have shown that amplified *MET* is a clinically valid therapeutic target for MET inhibitors or MET-tyrosine kinase inhibitors (e.g. crizotinib) approved recently for the treatment of patients with NSCLC [9].

The frequency of *MET*amp as detected by FISH in NSCLC is variable in the literature, ranging from 3% to 10%. This range in frequency is likely attributable to a lack of standardization of FISH techniques, different cutoffs for defining *MET* positivity, and/or patient selection criteria across studies [6, 10–16]. For example, in a study of 213 Asian patients with NSCLC, Okuda et al. [16] used a cutoff of 3 or more copies of *MET* per cell to define *MET*-FISH positivity. They reported that patients with *MET*-positive tumors had a shorter OS than did patients with a normal *MET*-FISH result. In contrast, Cappuzzo et al. [6] used a cutoff of 5 copies per cell to designate *MET*-FISH positivity because patients with 5 or more copies of *MET* showed a worse clinical outcomes than did those with fewer than 5 copies. In another study involving 141 patients with stage I lung adenocarcinoma, a mean *MET* copy number (*MET*-CN) ≥ 3.4 per cell was regarded as a positive result and was associated with a poorer prognosis [3]. In a recent study, Noonan and colleagues [17] proposed using a combination of criteria to establish *MET*-FISH positivity: a *MET*/CEP7 (centromeric probe of chromosome 7) ratio of ≥ 1.8 or a *MET*-CN ≥ 5. The variations among these studies indicate that the criteria used to define *MET*-FISH positivity are inconsistent and that researchers need to reach a consensus on a positive cutoff point above which *MET*-CN has a clinical impact on outcomes in patients with NSCLC [6, 10–16].

Although preclinical studies have shown that tumor cells with *MET*amp display significantly increased sensitivity to MET inhibitors or MET tyrosine kinase inhibitors (MET-TKIs) [18–20], several clinical trials of MET inhibitors have failed in patients with *MET*amp as determined by FISH [21–23]. One potential reason for the failure of these trials may be that *MET*-CN status cannot be assessed consistently or accurately because no standard *MET*-FISH reporting criteria is available. In addition, most clinical studies related to *MET*-CN status were conducted in NSCLC patients who had undergone surgical resection; very limited data are available on patients with unresectable stage IV disease [3, 6, 16]. Although one recent study population included about 60% stage IV patients, no detailed clinical data were provided [17].

To improve patient selection criteria for trials of targeted MET inhibitors, we conducted a comprehensive retrospective review of *MET*-FISH data of NSCLC patients evaluated during a 6-year period. We evaluated and validated *MET*-FISH reporting criteria proposed in the literature and correlated *MET*-FISH results with clinical outcome and disease progression in NSCLC patients who presented without distant metastasis. We also used these data to suggest more optimized *MET*-FISH reporting criteria to facilitate accurate determination of clinically relevant *MET*-CN status and improve risk stratification of patients with NSCLC.

## RESULTS

### Clinicopathologic data

The study group included 384 NSCLC specimens tested for *MET*-CN. The patient cohort consisted of 184 (47.9%) men and 200 (52.1%) women, with a median age of 64 years (range, 31–89 years). More than three quarters of the patients (305, 79.4%) were white. Adenocarcinoma accounted for 338 (88%) cases and squamous cell carcinoma accounted for 40 (10.4%) cases. The cohort consisted of 42 (10.9%) patients with stage I NSCLC, 42 (10.9%) with stage II disease, 86 (22.4%) with stage III disease, and 214 (55.7%) with stage IV disease. All patients received the standard-of-care therapy according to their disease stage at diagnosis, including but not limited to surgery, chemotherapy, radiation therapy, or epidermal growth factor receptor (EGFR) inhibitor therapy when applicable. No patients were treated with MET inhibitors. Table 1 provides detailed clinicopathological and demographic data summarized by disease stage. Stages I, II, III were grouped together because most patients who do not have distant metastasis are considered clinically eligible for surgery, whereas patients with stage IV disease are not candidates for surgery.

**Table 1:** Patient demographic and clinicopathologic characteristics

Characteristic	Total	Stages I-III	Stage IV	*P* value
	(n =384)	(n =170)	(n =214)	
Median age, years (range)	64 (31–89)	65 (32–88)	62 (31–89)	.25
Sex, n (%)				.751
Male	184 (47.9)	83 (48.8)	101 (47.2)	
Female	200 (52.1)	87 (51.2)	113 (52.8)	
Race/ethnicity, n (%)				.463
White	305 (79.4)	138 (81.2)	167 (78.0)	
Black	25 (6.5)	11 (6.5)	14 (6.5)	
Hispanic	24 (6.3)	12 (7.1)	12 (5.6)	
Asian	23 (6.0)	6 (3.5)	17 (7.9)	
Unknown	7 (1.8)	3 (1.8)	4 (1.9)	
Histology^a^, n (%)				.002^*^
Adenocarcinoma	338 (88.0)	139 (81.8)	199 (93.0)	
Squamous cell carcinoma	40 (10.4)	26 (15.3)	14 (6.5)	
Other	6 (1.6)	5 (2.9)	1 (0.5)	

^a^ Other histology included 5 patients with unclassified non-small cell lung cancer and 1 patient with adenosquamous carcinoma.

^*^Indicates statistically significant result (*P* <.05).

### Optimization of *MET*-FISH reporting criteria

Patients were first stratified into 5 groups according to *MET*-CN and *MET*/CEP7 ratio (Groups 1-4 *MET*/CEP7 ratio < 2.0) and: Group 1 characterized by *MET*-CN < 3 (273 patients, 71.1%); Group 2 with *MET*-CN ≥ 3 to < 4 (65 patients, 16.9%); Group 3 characterized by *MET*-CN ≥ 4 to < 5 (17 patients, 4.4%); Group 4 with *MET*-CN ≥ 5 (8 patients, 2.1%); and Group 5 in which the *MET*/CEP7 ratio ≥ 2.0 or signal clusters seen in > 10% of tumor cells (21 patients, 5.5%) (Supplementary Table 1) [24].

**Figure 1:**
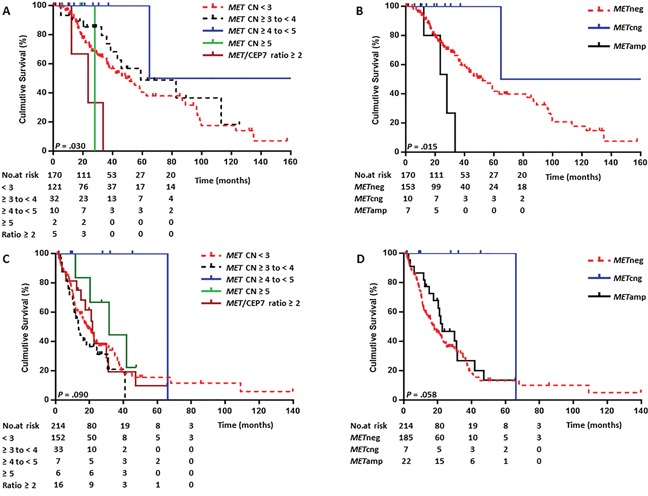
Kaplan-Meier curves comparing overall survival (OS) by *MET* FISH status **(A)** Comparison of OS in patients with stage I-III NSCLC stratified into 5 *MET*-CN groups. **(B)** Comparison of OS in the stage I-III NSCLC patients using the optimized *MET*-FISH reporting criteria. **(C)** Comparison of OS in patients with stage IV NSCLC stratified into 5 *MET*-CN groups. **(D)** Comparison of OS in patients with stage IV NSCLC using the optimized *MET*-FISH reporting criteria.

When we tested for associations between these groups and OS in patients with stages I to III disease, we found that patients in Groups 1 and 2 had a similar median OS (48.9 months and 59.1 months, respectively; *P* =.321). Groups 4 and 5 also had similar OS (28.1 months vs. 23.6 months, respectively; *P* =.782). Patients in Groups 1 and 2 had markedly longer median OS than did patients in Groups 4 and 5. Interestingly, patients in Group 3 had significantly longer median OS than patients in any other group (134.4 months; *P* =.03) (Figure 1A and Supplementary Table 2).

Because our OS data for patients with stages I-III disease were consistent with those of reported previously in a large study [6], we next re-categorized the data using a *MET*-CN cutoff of 5 copies and a *MET/*CEP7 ratio cutoff of 2.0. These optimized *MET*-FISH reporting categories were as follows:

– *MET*amp: (A) *MET*/CEP7 ratio ≥ 2.0, or (B) *MET*-CN ≥ 5, or (C) *MET*/CEP7 ratio < 2.0 but *MET*-CN ≥ 20 or *MET* signal clusters in more than10% of tumor cells.
– *MET*-CN-gain (*MET*cng): *MET*/CEP7 ratio < 2.0 and *MET*-CN ≥ 4 to < 5.
– *MET*neg: *MET*/CEP7 ratio < 2.0 and *MET*-CN < 4.

We reclassified all cases into these 3 categories for further analyses of OS and risk stratification (Figure 1B and Supplementary Table 2).

### *MET*amp is highly associated with stage IV NSCLC

The mean *MET*-CN in the overall study cohort was 3.1 copies per cell (range, 1.5 - 20.5). The mean *MET*/CEP7 ratio was 1.15 (range, 0.5 - 8.5). The mean *MET*-CN and mean *MET*/CEP7 ratio were significantly higher in patients with stage IV NSCLC than in patients with stages I-III disease (*P* =.042 and *P* =.016, respectively) (Supplementary Table 3). Using the optimized *MET*-FISH reporting criteria, 29 of 384 (7.6%) patients were categorized as having *MET*amp.

Among the 170 patients with stage I-III disease, 153 (90%) were in the *MET*neg group, 10 (5.9%) in the *MET*cng group, and 7 (4.1%) in the *MET*amp group. Of the 214 patients with stage IV disease, 185 (86.4%) were in the *MET*neg group and 7 (3.3%) in the *MET*cng group. We determined that 22 (10.3%) patients with stage IV NSCLC had *MET*amp, a significantly higher rate than that found in patients with stages I-III disease (*P* =.038), indicating that *MET*amp was highly associated with advanced disease stage (Supplementary Table 3 and Table 2).

**Table 2:** Demographic and clinical characteristics of 384 patients in the three optimized *MET* categories

Characteristic	Total (n = 384)	*MET*neg (n = 338)	*MET*cng (n = 17)	*MET*amp (n = 29)	*P* value
Median age at diagnosis (years)	63	64	62	61	.338
Sex, n (%)					.549
Male	184 (47.9)	165 (48.8)	8 (47.1)	11 (37.9)	
Female	200 (52.1)	173 (51.2)	9 (52.9)	18 (62.1)	
Race/ethnicity, n (%)					1.000
White	305 (80.9)	267 (80.7)	14 (82.4)	24 (82.8)	
Non-white^a^	72 (19.1)	64 (19.3)	3 (17.6)	5 (17.2)	
Histology, n (%)					.364
Adenocarcinoma	338 (88.0)	296 (87.6)	17 (100.0)	25 (86.2)	
Non-adenocarcinoma^b^	46 (12.0)	42 (12.4)	0 (0.0)	4 (13.8)	
Stage, n (%)					.064
I	42 (10.9)	37 (10.9)	4 (23.5)	1 (3.4)	
II	42 (10.9)	39 (11.5)	0 (0.0)	3 (10.3)	
III	86 (22.4)	77 (22.8)	6 (35.3)	3 (10.3)	
IV	214 (55.7)	185 (54.7)	7 (41.2)	22 (75.9)	
Distant metastasis^c^ (%)					.038^*^
No	170 (44.3)	153 (45.3)	10 (58.8)	7 (24.1)	
Yes	214 (55.7)	185 (54.7)	7 (41.2)	22 (75.9)	

Abbreviations: *MET*, mesenchymal-epithelial transition factor; FISH, fluorescence *in situ* hybridization; *MET*neg, *MET*-negative; *MET*cng, *MET* copy number gain; *MET*amp, *MET* amplification.

^a^Non-white included 25 black, 24 Hispanic, and 23 Asian patients. Seven patients with unknown race were excluded from the analysis.

^b^Non-adenocarcinoma included 40 patients with squamous cell carcinoma, 5 patients with unclassified non-small cell lung cancer, and 1 patient with adenosquamous carcinoma.

^c^Distant metastasis included bone, brain, liver, adrenal, contralateral lobe, tumor with pleural nodules, or malignant pleural effusion.

^*^ Indicates statistically significant result (*P* <.05, Fisher exact test).

### *MET*-FISH and MET-IHC correlations

MET overexpression as assessed by immunohistochemistry (IHC) was available in 202 of 384 (52.6%) patients, including 92 of 170 (54.1%) patients with stages I-III and 110 of 214 (51.4%) patients with stage IV disease. MET overexpression was found in 51 (55.4%) patients in stages I-III and 73 (66.4%) patients with stage IV (*P* =.14), indicating that MET overexpression is not associated significantly with disease stage (Supplementary Table 5). In addition, when we correlated MET expression with *MET*-CN, 20 of 122 *MET*neg cases, 3 of 8 *MET*cng cases, and 5 of 22 *MET*amp cases were MET-IHC negative (*P* =.28) (Supplementary Table 5). The overall concordance between the *MET*-FISH and the MET-IHC was 44.8% (*P* =.11) (Supplementary Table 5).

### METamp is associated with shorter OS in patients with stages I-III NSCLC

The median follow-up duration was 19.5 months and the median OS was 36.4 months for the overall study cohort. OS was not associated with patient age (*P* =.18), sex (*P* =.58), or race/ethnicity (*P* =.45). As expected, longer OS was highly associated with early-stage (I-III) disease (*P* <.001) and with adenocarcinoma (*P* <.001) (Supplementary Table 4).

Under the optimized *MET*-FISH reporting criteria, the median OS durations of early-stage patients with *MET*amp, *MET*cng, and *MET*neg were 28.1, 134.4, and 51.6 months, respectively. Patients with *MET*amp had significantly shorter OS than did patients with *MET*neg (*P* =.036), and patients with *MET*cng had longer OS than did patients with *MET*neg, although the difference was not statistically significant (*P* =.109) (Figure 1B, Table 3)

**Table 3:** Univariate and multivariate analyses of overall survival and progression to distant metastasis for patients with stages I-III NSCLC

Variable		Overall Survival						Progression to Distant Metastasis^a^					
		Univariate			Multivariate			Univariate			Multivariate		
		HR	95% CI	*P* value	HR	95% CI	*P* value	HR	95% CI	*P* value	HR	95% CI	*P* value
Age	> 64 years	-	-	-	-	-	-	-	-	-	-	-	-
	≤ 64 years	0.65	0.41-1.03	.066	0.75	0.46-1.22	.243	1.24	0.79-1.96	.343	1.24	0.78-1.98	.372
Sex	Female	-	-	-	-	-	-	-	-	-	-	-	-
	Male	1.39	0.88-2.18	.159	1.03	0.64-1.67	.89	1.34	0.86-2.11	.197	1.19	0.75-1.90	.467
Race^b^	Non-white	-	-	-	-	-	-	-	-	-	-	-	-
	White	2.05	0.98-4.29	.055	2.05	0.93-4.48	.074	1.2	0.64-2.22	.572	1.43	0.75-2.76	.281
Histology^c^	Non-ADC	-	-	-	-	-	-	-	-	-	-	-	-
	ADC	0.34	0.20-0.57	<.001^*^	0.52	0.30-0.90	.019^*^	0.85	0.47-1.55	.593	0.97	0.52-1.81	.929
Stage	I	-	-	-	-	-	-	-	-	-	-	-	-
	II	1.57	0.78-3.17	.206	1.38	0.67-2.84	.377	0.95	0.47-1.92	.88	0.92	0.45-1.87	.807
	III	3.28	1.77-6.10	<.001^*^	3.33	1.73-6.42	<.001^*^	2.21	1.25-3.91	.007^*^	2.25	1.24-4.09	.008^*^
*MET* FISH	*MET*neg	-	-	-	-	-	-	-	-	-	-	-	-
	*MET*cng	0.2	0.03-1.43	.109	0.24	0.03-1.79	.165	0.93	0.34-2.55	.883	0.93	0.33-2.60	.894
	*MET*amp	3.01	1.08-8.42	.036^*^	3.26	1.15-9.23	.026^*^	4.25	1.67-10.81	.002^*^	4.86	1.85-12.75	.001^*^

Abbreviations: HR, hazard ratio; CI, confidence interval; *MET*, mesenchymal-epithelial transition factor; FISH, fluorescence *in situ* hybridization; *MET*neg, *MET*-negative; *MET*cng, *MET* copy number gain; *MET*amp, *MET* amplification; hyphens (-) indicate the reference category. ADC: Adenocarcinoma.

^a^Distant metastasis included bone, brain, liver, adrenal, contralateral lobe, tumor with pleural nodules, or malignant pleural effusion.

^b^Non-white included 11 black, 12 Hispanic, and 6 Asian patients. Three patients with unknown race were not included in the analysis.

^c^The adenocarcinoma category also included bronchoalveolar histology. Non-adenocarcinoma included 26 patients with squamous cell carcinoma, 4 patients with unclassified non-small cell lung cancer, and 1 patient with adenosquamous carcinoma.

^*^ Indicates statistically significant result (*P* <.05).

In patients with stage IV tumors, *MET*-CN status showed no statistically significant association with OS (Figures 1C and 1D, Table 4). However, patients with *MET*cng did have substantially longer OS than did patients with *MET*neg (66.2 vs. 17.5 months, *P* =.053). This pattern was similar to that observed in patients with stages I-III disease.

**Table 4:** Univariate and multivariate analyses of overall survival for patients with stage IV NSCLC

Variable		Univariate			Multivariate		
		HR	95% CI	*P* value	HR	95% CI	*P* value
Age	> 64 years	-	-	-	-	-	-
	≤ 64 years	0.77	0.54-1.09	.141	0.81	0.56-1.15	.239
Sex	Male	-	-	-	-	-	-
	Female	0.88	0.62-1.25	.485	0.83	0.58-1.19	.315
Race^a^	Non-white	-	-	-	-	-	-
	White	0.95	0.62-1.45	.803	0.98	0.64-1.52	.938
Histology^b^	Non-adenocarcinoma	-	-	-	-	-	-
	Adenocarcinoma	0.62	0.34-1.12	.115	0.59	0.32-1.09	.095
*MET*-FISH	*MET*neg	-	-	-	-	-	-
	*MET*cng	0.14	0.02-1.02	.053	0.17	0.02-1.20	.075
	*MET*amp	0.79	0.46-1.36	.374	0.79	0.46-1.36	.400

Abbreviations: HR, hazard ratio; CI, confidence interval; *MET*, mesenchymal-epithelial transition factor; FISH, fluorescence *in situ* hybridization; *MET*neg, *MET*-negative; *MET*cng, *MET* copy number gain; *MET*amp, *MET* amplification; hyphens (-) indicate the reference category.

^a^ Non-white included 14 black, 12 Hispanic, and 17 Asian patients. Four patients with unknown race were not included in the analysis.

^b^ The adenocarcinoma category also included bronchoalveolar histology. Non-adenocarcinoma included 14 patients with squamous cell carcinoma and 1 patient with unclassified non-small cell lung cancer.

### METamp is associated with earlier distant metastases in patients with stages I-III NSCLC

**Figure 2:**
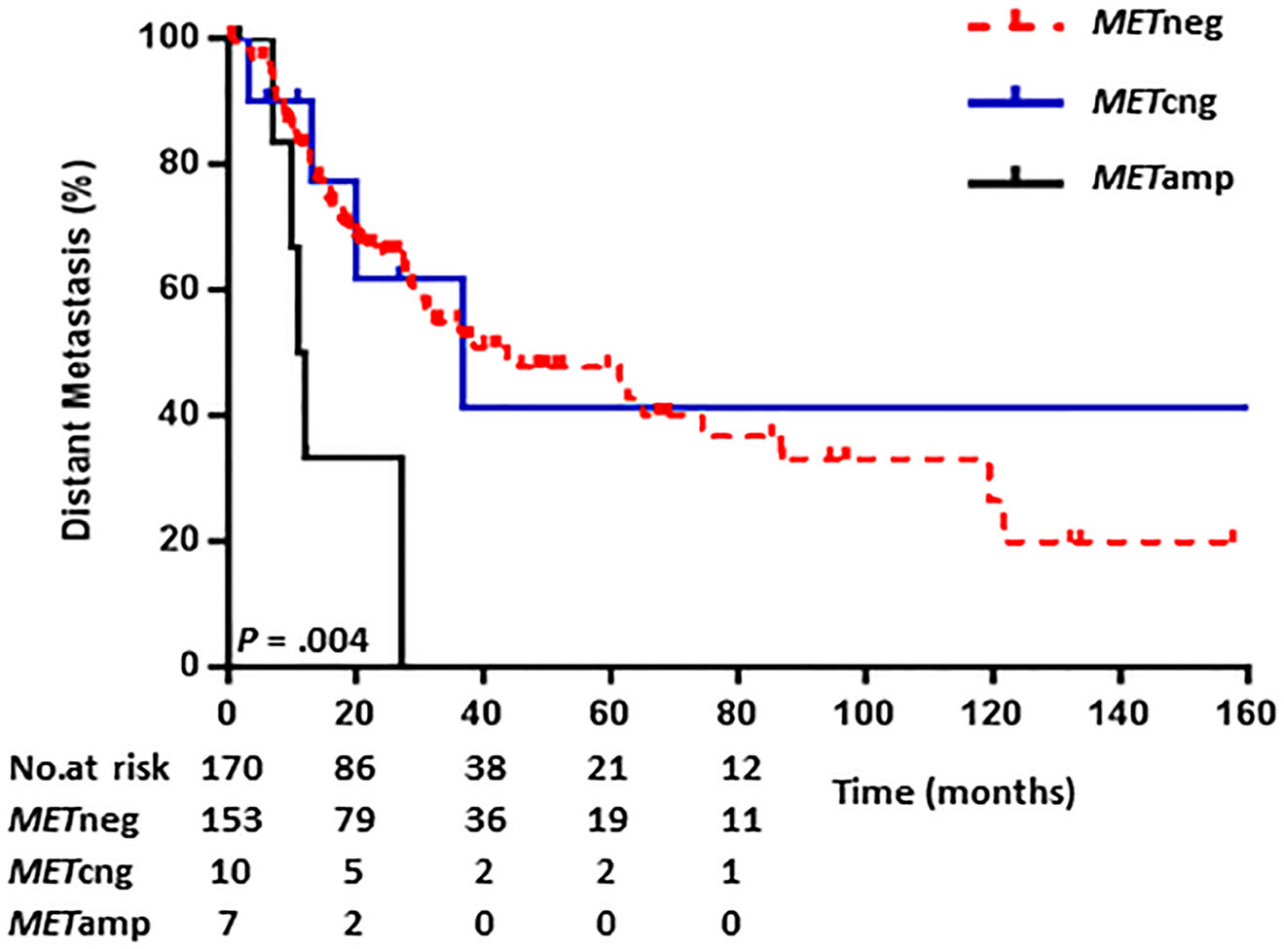
Kaplan-Meier curves comparing time to distant metastasis using the optimized reporting criteria Note: Distant metastasis included bone, brain, liver, adrenal, contralateral lobe, tumor with pleural nodules, or malignant pleural effusion.

We assessed the relationship between *MET*-CN and disease metastases in patients with stages I-III NSCLC. The 7 patients with *MET*amp tumors had a significantly shorter median time to distant metastasis (11.6 months) than did patients with *MET*neg (43.8 months; *P* =.004) or *MET*cng (37.0 months) tumors (Table 3 and Figure 2), indicating that *MET*amp is a risk factor for earlier distant metastases in patients with NSCLC.

### *MET*amp is an independent predictor of worse OS in patients with stages I-III NSCLC

Multivariate analyses revealed among patients with stages I-III tumors that patients with adenocarcinoma had longer OS than did patients with other tumor types (hazard ratio [HR], 0.85; 95% confidence interval [CI], 0.30 - 0.90; *P* =.019). Patients with stage III tumors showed a higher risk for distant metastases (HR, 2.25; 95% CI, 1.24 - 4.09; *P* =.008) and for shorter OS (HR, 3.33; 95% CI, 1.73–6.42; *P* <.001) than did patients with stage I disease. Patients with *MET*amp tumors had significantly shorter OS than did patients with *MET*neg tumors (HR, 3.26; 95% CI, 1.15 - 9.23; *P* =.026). In addition, *MET*amp was found to be highly associated with distant metastases (HR, 4.86; 95% CI, 1.85 - 12.75; *P* =.001) (Table 3 and Figure 2). However, in patients with stage IV disease, *MET*amp showed no significant impact on outcomes in either the univariate or the multivariate analyses (Table 4).

### MET overexpression assessed by IHC is not associated with clinical outcomes

Of 202 patients with MET-IHC data available, the median OS was 24.5 months in patients with MET overexpression versus 36.5 months observed in patients with a normal MET expression (*P* =.31). We further assessed the potential clinical impact of MET overexpression in patients with different disease stages. In 92 patients with stage I-III disease, the median OS was 51.6 months in those with tumors with normal MET expression (n = 41) versus 54.9 months in patients with tumors with MET overexpression (n = 51) (*P* =.97). In 110 patients with stage IV disease, the median OS was 16.3 months in those with tumors that showed normal MET expression (n = 37) versus 20.4 months in those patients with tumors with MET overexpression (n = 73) (*P* =.67) (Supplementary Figure 1). Our data indicate that MET overexpression alone does not impact clinical outcome.

## DISCUSSION

In this study, we evaluated data on *MET*-CN status in 384 NSCLC patients with various disease stages and identified associations between *MET*-CN and clinical outcome. To resolve the conflicting *MET*-FISH reporting criteria in the literature, we stratified our patient cohort into 5 groups based on *MET*-CN and *MET*/CEP7 ratio and identified associations between these groups and outcomes. We focused initially on patients with stage I-III tumors because *MET*amp has been reported to be associated with stage IV tumors [6]. On the basis of overall survival, we optimized our *MET*-FISH reporting criteria by integrating *MET*-CN and *MET*/CEP7 ratio and reclassifying the patients into 3 groups: *MET*amp, *MET*cng, and *MET*neg. These new, optimized criteria merged groups 4 and 5 (*MET*-CN ≥ 5 and *MET/*CEP7 ratio ≥ 2.0) into one group, *MET*amp, because they had similar OS (Supplementary Table 1). These results were consistent with previous reports [6]. Moreover, patients with *MET*cng (*MET*/CEP7 ratio < 2.0 and *MET*-CN *≥* 4 to < 5) had markedly better OS than did patients in the other groups, suggesting that patients with *MET*cng constitute a distinct group of NSCLC patients. We also found that patients with *MET*amp had a higher risk of distant metastasis than did patients in the other groups.

According to the *MET*-FISH reporting criteria we suggest, the overall frequency of *MET*amp in this study cohort was about 8%. We noted that *MET*amp was significantly more common in patients with stage IV disease than in patients without distant metastases. We confirmed that *MET*amp was an independent risk factor for poor OS in patients with early-stage (I-III) NSCLC (28.1 months vs. 134.35 months in *MET*cng and 51.6 months in *MET*neg, *P* =.015), consistent with results reported previously [6]. Importantly, all patients with *MET*amp and stage I-III disease in this study cohort developed distant metastases within 1 year of diagnosis, indicating that *MET*amp status predicts early distant metastases in patients with stages I-III NSCLC. Although 34 patients in the stage I-III disease group were positive for *EGFR* mutation, when this subset was compared with those who had no *EGFR* mutations, there was no significant impact observed on OS or progression to distant metastasis.

Only limited clinical data have been published on *MET*-CN status and its clinical implications in patients with stage IV NSCLC [3, 6, 16]. In this study, 214 patients with stage IV disease were investigated. We found that *MET*amp was highly associated with stage IV NSCLC, but did not further negatively impact clinical outcomes when compared with the *MET*neg category for patients with the same disease stage. However, we found that patients with stage IV disease and *MET*cng had much longer OS durations than did those with either *MET*neg or *MET*amp (66.2 months vs. 17.5 months in *MET*neg vs. 22.8 months in *MET*amp, *P* =.053).

The *MET*cng category identified in this cohort likely represents a distinct group of NSCLC patients, similar to that identified by Cappuzzo et al. [6], although that study classified patients with *MET*-CN ≥ 4 to < 5 as *MET*neg and for that reason did not provide full clinical data for all patients. We speculate that the *MET*cng category may represent patients with multiple polysomies e.g. near-tetrasomies as indicated by the copy numbers. Most of the 17 *MET*cng patients in this cohort, who presumably had near-tetrasomy of chromosome 7, also showed evidence of having near-tetrasomies of chromosomes 2 (by *ALK*-FISH), 6 (by *ROS1-*FISH), or 10 (by *RET*-FISH) (data not shown). *MET*cng coinciding with CN gains of *ALK, ROS1*, and *RET* likely resulted from genome-wide polysomies, particularly near-tetraploidy. Similar findings have been reported in a study of 47 patients with *EGFR*-negative lung cancer, in which 13 patients with polysomy 7 showed improved progression-free survival [25]. To further confirm the FISH findings, we performed OncoScan microarray (Affymetrix) on a subset of patients based on the availability of FFPE tumor blocks including 5 *MET*cng samples. SNP array data showed an overall good concordance with FISH, and the normalized genomic profiles in cases with *MET*cng showed significantly lower genomic copy number complexity comparing the *MET*neg and *MET*amp groups. Although our microarray sample number was small, such *MET*cng cases with a low frequency of somatic copy number alterations (SCNAs) [1, 2] could be clinically less aggressive and likely associated with a better outcome, however, the underline mechanisms remain to be explored and a genome wide assessment of SCNAs in a large study is needed to confirm our findings.

On the basis of these observations, we argue against treating patients who have *MET*cng with MET inhibitors. Several clinical trials have failed to show any positive effect of MET inhibitors on patient survival. We speculate that these studies may have inaccurately identified patients with *MET*cng as having *MET*amp. For example, in one phase II study of 37 *MET*-positive patients [23], *MET* positivity was defined as *MET*-CN ≥ 4 in over 40% of cells; only 3 of the 37 patients with a *MET*/CEP7 ratio > 2.0 would have been interpreted as having *MET*amp using the criteria we suggest in this study. Similarly, in a phase III study, only 4 of 54 patients had a *MET*/CEP7 ratio > 2.0 [22]. We speculate that the patients enrolled in these two trials may have had *MET*cng, not “real” *MET*amp, and that this may explain the poorer response among these patients to MET inhibitors. Our results highlight the clinical importance of accurate *MET*-CN assessment using standardized and optimized reporting criteria in determining eligibility for clinical trials of MET inhibitors.

Cases with a *MET*-CN at the borderline of *MET*neg and *MET*cng (3.8-3.9) or of *MET*cng and *MET*amp (4.8-4.9) can be challenging for risk stratification, as some of these results could be explained by tumor heterogeneity which are not uncommon in many cancers. However, in such cases, the overall estimated copy numbers i.e. by FISH for the assessment of oncogene amplifications are often underestimated [26]. Using the definition of *MET*amp in this study, the results are in line with what have been reported. In addition, using our suggested reporting criteria, those cases with tumor cells with clustering *MET*-FISH signals in > 10% cells would be considered to be positive and this can be considered as alternative method to address tumor heterogeneity to at least some degree. To further address tumor heterogeneity, we recommend reflex testing by repeating FISH or reflex testing using alternative assays, such as chromogenic *in situ* hybridization [27], microarray-based technology [28, 29], comprehensive molecular characterizations or sequencing based single cell analysis, etc. to accurately determine relevant copy numbers in cells with tumor heterogeneity as previously reported [1, 2, 30]. We are aware of that FISH based testing i.e. the *MET*-FISH cannot assess genome-wide SCNAs that are often associated with treatment resistance or disease progression in NSCLS, and an integrated genomic approach to accurately assess SCNAs could be utilized in the clinical settings in near future [1, 2].

Over half of the patients in the current cohort were also assessed for MET expression status by IHC. Our results show that MET overexpression were not associated with disease stages, although patients with *MET*amp did have the higher number of cases with MET overexpression compared with the *MET*neg and *MET*cng subgroups (Supplementary Table 5). Unlike the ideal concordance observed *HER2* amplification and HER2 overexpression reported previously [31] the overall concordance between *MET*-CN and MET expression observed in this study was low (~ 45%) which was consistent with what has been reported by others [32]. Similar discordance also has been observed in gastric-intestinal cancer studies by tissue microarray (unpublished data). In addition, the clinical impact of MET overexpression has been controversial, with some studies showing negative impact [33] and others showing the opposite results [32]. Our data did not show independent impact by MET overexpression alone assessed by IHC. We propose that standardized IHC reporting criteria is also needed [34] and that more studies should be conducted to determine if MET protein overexpression assessed by IHC alone is more informative than the *MET*-CN by FISH [33].

In conclusion, we suggest that the optimized *MET*-FISH reporting criteria that are useful for stratifying risk in patients with all stages and histological types of NSCLC. Our results indicated that *MET*amp is strongly associated with stage IV tumors and that *MET*-FISH testing can identify patients who are eligible for treatment with MET inhibitors [35]. Furthermore, *MET*amp is an independent predictor of poorer prognosis in patients with stages I-III NSCLC. *MET*amp is also a reliable predictor of distant metastases in patients with early stages NSCLC and, such patients may be candidates for more intensive treatments, such as combination therapy using MET inhibitors after surgery. Finally, we found that *MET*cng could represent an independent prognostic group of patients with NSCLC; however, more data are needed to confirm this observation.

## PATIENTS AND METHODS

### Patients

We retrospectively reviewed the records of all patients with NSCLC who had been tested for *MET* using FISH between February 1, 2010 and December 31, 2015 at MD Anderson Cancer Center. All patients diagnosed with NSCLC for whose detailed clinicopathologic data are available were included. Patients with ALK receptor tyrosine kinase (*ALK*), ROS proto-oncogene 1 receptor tyrosine kinase (*ROS1*), or ret proto-oncogene (*RET*) gene rearrangements were excluded from the study. Clinical disease stage for all patients was determined using the National Comprehensive Cancer Network staging system for NSCLC [36]. This study was approved by the Institutional Review Board.

### FISH analysis

*MET*-copy-number (*MET*-CN) status was assessed using a dual-color FISH probe set (CymoGenDX, Irvine, CA) targeting *MET* and CEP7on formalin-fixed, paraffin-embedded tissue sections from tumor specimens following established standard laboratory procedures. *MET*-CN and the number of CEP7 per nucleus were scored in 60 cells and the mean *MET*/CEP7 ratio was calculated for each specimen. A subset of FFPE tissue specimens from at least 20 normal individuals was included to establish normal cutoff values following laboratory standard procedures (e.g. chromosome 7 aneuploidy such as monosomy or trisomy/tetrasomy/polysomy 7). The initial reporting criteria used at our institution during the study period, which we designate here as “historical” *MET*-FISH reporting criteria, classified cases into two categories, *MET*amp and MET-negative (*MET*neg), with a cutoff of *MET*/CEP7 ratio of 2.0. A sample was considered to have *MET*amp if the mean *MET*/CEP7 ratio was ≥ 2.0 or if the *MET*/CEP7 ratio was < 2.0 but the *MET*-CN was ≥ 20 copies/cell or *MET* signal clusters were seen in more than10% of tumor cells [24].

### Immunohistochemistry (IHC) analysis

MET immunohistochemical staining (MET-IHC) was evaluated using a Benchmark Ultra Autostainer (Ventana, Tucson, AZ) with anti-total c-MET (SP44) rabbit monoclonal primary antibody (Ventana, Tucson, AZ), following the manufacturer’s instructions. Staining was scored by determining the percentage of cells showing weak (1+), moderate (2+), or strong (3+) membranous staining. MET overexpression was considered as positive if ≥ 50% of tumor cells showing cellular membrane staining at an intensity of “2+” or “3+” [37]. Scoring was performed independently by two individuals and any discrepant cases were re-evaluated for the final interpretation.

### Statistical analysis

Patient and tumor characteristics, including demographics, tumor type, and *MET*-CN, were summarized using frequencies, percentages, and distributions. Categorical variables were compared using chi-square and Fisher exact tests, and continuous variables were compared using the Student *t* test. OS was calculated from the date of first diagnosis to the date of last follow-up or death of the patient. The log-rank test and Kaplan-Meier curves were employed to compare OS between subgroups. GraphPad Prism software version 6 (GraphPad Software, La Jolla, CA) was used for the survival analyses. Multivariable analyses, including Cox proportional hazards regression analysis, were performed using SPSS software version 9.3 (SPSS, Inc., Chicago, IL). All tests were 2-sided when appropriate, and differences were considered significant at *P* <.05.

## SUPPLEMENTARY MATERIALS FIGURE AND TABLES



## Abbreviations

NSCLC: Non-small cell lung cancer
*MET*: mesenchymal-epithelial transition factor
*MET*-CN: *MET* copy number
*MET*neg: *MET* negative
*MET*cng: *MET* copy number gain
*MET*amp: *MET* amplification
FISH: fluorescence *in situ* hybridization
IHC: immunohistochemistry
OS: overall survival
HR: hazard ratio
CI: confidence interval
