# Supplementary material for: MET amplification assessed using optimized FISH reporting criteria predicts early distant metastasis in patients with non-small cell lung cancer
Source: Oncotarget. 2018 Feb 7;9(16):12959–70. doi: 10.18632/oncotarget.24430 (PMC5849187; doi:10.18632/oncotarget.24430)
Supplement: Supplementary file 1 [file oncotarget-09-12959-s001.pdf]

MET amplification assessed using optimized FISH reporting criteria predicts early distant metastasis in patients with non-small cell lung cancer

SUPPLEMENTARY MATERIALS

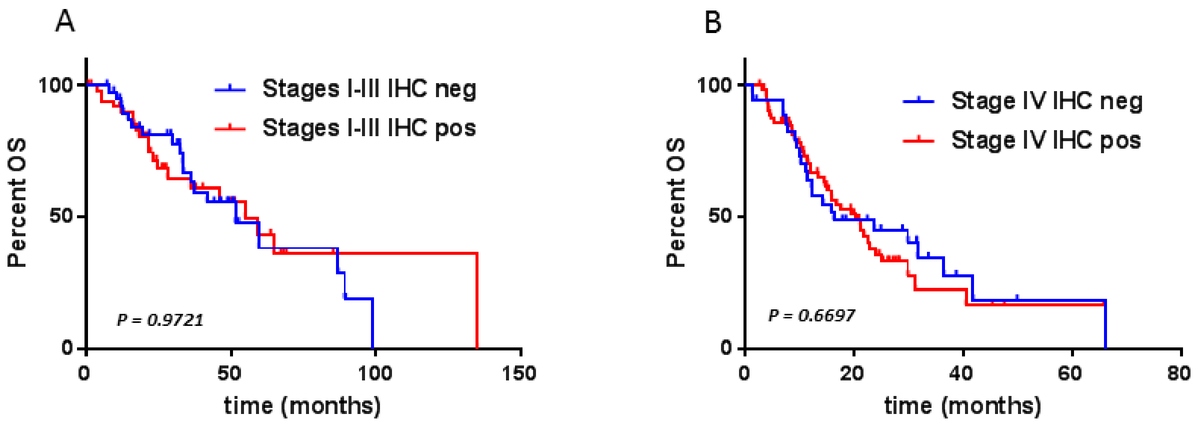

Supplementary Figure 1: Kaplan-Meier curves comparing overall survival (OS) by MET IHC status. **(A)** Comparison of OS in patients with (pos, positive) and without (neg, negative) MET overexpression in the stages I-III NSCLC group; **(B)** Comparison of OS in patients with (pos, positive) and without (neg, negative) MET overexpression in the stage IV NSCLC group.

Supplementary Table 1: *MET* copy number distribution according to optimized and historic criteria*

	No. of patients (%) (N = 384)		
**Optimized reporting criteria**	***MET*neg**	***MET*cng**	***MET*amp**
	338 (88.0)	17 (4.4)	29 (7.6)
**Historic reporting criteria**			
MET-negative			
*MET*CN< 3	273 (71.1)	0	0
*MET*CN≥ 3 to < 4	65 (16.9)	0	0
*MET*CN≥ 4 to < 5	0	17 (4.4)	0
*MET*CN ≥ 5	0	0	8 (2.1)
MET-positive			
MET/CEP7 ratio ≥ 2.0	0	0	21 (5.5)

Abbreviations: *MET*, mesenchymal-epithelial transition factor; *MET* CN: *MET* copy number per cell, *MET*neg, *MET*-negative; *MET*cng, *MET* copy number gain; *MET*amp, *MET* amplification; CEP7, centromere probe of chromosome 7.
*Both reporting criterion included those with "the *MET*/CEP7 ratio was < 2.0 but the *MET*-CN was ≥ 20 copies/cell or *MET* signal clusters were seen in more than10% of tumor cells".

Supplementary Table 2: Comparison of overall survival by *MET* status in patients with stages I-III NSCLC

Five-Group *MET* Classification	No. of Patients	OS1[a] (Months)	P1[b]	P2[c]	Optimized *MET* Categories	OS2[d] (Months)	P3[e]
					*MET*neg	51.6	Ref.
MET CN < 3 (Group1)	121	48.9	.034*	Ref.			
*MET*CN≥ 3 to < 4 (Group2)	32	59.1	.005*	.321			
					*MET*cng	134.4	.106
*MET*CN≥ 4 to < 5 (Group3)	10	134.4	.003*	.063			
					*MET*amp	28.1	.036*
MET CN ≥ 5 (Group4)	2	28.1	.782	.623			
MET/CEP7 ratio ≥ 2.0 (Group5)	5	23.6	Ref.	.034*			

Abbreviations: *MET*, mesenchymal-epithelial transition factor; *MET* CN: *MET* copy number per cell; OS, overall survival; *MET*amp, *MET* amplification; METcng, *MET* copy number gain; *MET*neg, *MET*-negative; CEP7, centromere probe of chromosome 7.

[a]OS1: OS according to the 5-group *MET* classification.[b]P1: *P* value for the 5-group classification with *MET*/CEP7 ratio ≥ 2.0 as the reference category.

[c]P2:*P* value for the 5-group classification with *MET*CN < 3 as the reference category.

[d]OS2: OS according to the optimized *MET* categories.

[e]P3:*P* value in the optimized categories with *MET*neg as the reference category. * Indicates statistically significant result (*P*<.05).

Supplementary Table 3: Comparison of *MET* parameters by disease stage

***MET* Parameter**	**Overall**	**Stage I-III**	**Stage IV**	***P* Value**
MET FISH Quantity				
Mean *MET*-CN per cell (range)	3.13 (1.48-20.5)	2.83 (1.50-13.68)	3.36 (1.48-20.5)	.042*
Mean CEP7per cell (range)	2.65 (1.37-9.6)	2.64 (1.65-7.48)	2.67 (1.37-9.60)	.743
Mean *MET*/CEP7 ratio(range)	1.15 (0.5-8.5)	1.06 (0.50-2.28)	1.21 (0.68-8.50)	.016*
MET FISH Categories				.038*
*MET*neg, n (%)	338	153 (90.0)	185 (86.4)	
*MET*cng, n (%)	17	10 (5.9)	7 (3.3)	
*MET*amp, n (%)	29	7 (4.1)	22 (10.3)	

Abbreviations: *MET*, mesenchymal-epithelial transition factor; *MET* CN: *MET* copy number per cell; CEP7, centromere probe of chromosome 7; FISH, fluorescence *in situ* hybridization; *MET*neg, *MET*-negative; *MET*cng, *MET* copy number gain; *MET*amp, *MET* amplification. * Indicates statistically significant result (P <.05, Fisher exact test or *t* test).

Supplementary Table 4: Overall survival and patient clinicopathological characteristics

Characteristic	No. of Patients	Median OS (months)	*P* Value
Age (years)			.184
≤ 64	199	34.8	
> 64	185	30.9	
Sex			.575
Male	184	29.9	
Female	200	36.0	
Race/ethnicity			.450
White	305	32.1	
Non-white[a]	72	31.6	
Histology			<.001*
Adenocarcinoma	338	34.8	
Others[b]	46	24.7	
Stage			<.001*
I-III	170	51.6	
IV	214	19.5	

[a] Non-white included 25 black, 24 Hispanic, and 23 Asian patients. Seven patients with unknown race were excluded from the analysis.
[b] Others included 40 patients with squamous cell carcinoma, 5 patients with unclassified non-small cell lung cancer, and 1 patient with adenosquamous carcinoma.
* Indicates statistically significant result (P<.05).

Supplementary Table 5: MET protein overexpression in patients with different stages and *MET* copy number groups

	Total, N (%)	Stages, N (%)		*P* Value	*MET*-CN Status, N (%)			*P* Value
	N = 384	I-III<br>N = 170	IV<br>N = 214		*MET*neg<br>N = 338	*MET*cng<br>N = 17	*MET*amp<br>N = 29	
**MET-IHC**				***0.14***				***0.28***
negative	78 (20.3)	41 (24.1)	37 (17.3)		20 (20.7)	3 (17.7)	5 (17.2)	
positive	124 (32.3)	51 (30.0)	73 (34.1)		102 (30.2)	5 (29.4)	17 (58.6)	
not available	182 (47.4)	78 (45.9)	104 (48.6)		166 (49.1)	9 (52.9)	7 (24.1)	

Abbreviations: *MET*, mesenchymal-epithelial transition factor; *MET* CN: *MET* copy number per cell, *MET*neg, *MET*-negative; *MET*cng, *MET* copy number gain; *MET*amp, *MET* amplification; IHC, immunohistochemistry.
